# Emotion and memory: Event-related potential indices predictive for subsequent successful memory depend on the emotional mood state.

**DOI:** 10.2478/v10053-008-0001-8

**Published:** 2008-07-15

**Authors:** Markus Kiefer, Stefanie Schuch, Wolfram Schenck, Klaus Fiedler

**Affiliations:** 1 University of Ulm, Department of Psychiatry, Ulm, Germany; 2 University of Wales, Department of Psychology, Bangor, UK; 3 University of Bielefeld, Department of Technology, Bielefeld, Germany; 4 University of Heidelberg, Department of Psychology, Heidelberg, Germany

**Keywords:** episodic memory, emotion, cognitive styles, subsequent memory effect, event-related potentials

## Abstract

The present research investigated the influencesof emotional mood states on cognitive processes and neural circuits during long-term memory encoding using event-related potentials (ERPs). We assessed whether the subsequent memory effect (SME), an electrophysiological index of successful memory encoding, varies as a function of participants’ current mood state. ERPs were recorded while participants in good or bad mood states were presented with words that had to be memorized for subsequent recall. In contrast to participants in bad mood, participants in good mood most frequently applied elaborative encoding styles. At the neurophysiological level, ERP analyses showed that potentials to subsequently recalled words were more positive than to forgotten words at central electrodes in the time interval of 500-650 ms after stimulus onset (SME). At fronto-central electrodes, a polarity-reversed SME was obtained. The strongest modulations of the SME by participants’ mood state were obtained at fronto-temporal electrodes. These differences in the scalp topography of the SME suggest that successful recall relies on partially separable neural circuits for good and bad mood states. The results are consistent with theoretical accounts of the interface between emotion and cognition that propose mood-dependent cognitive styles.

## INTRODUCTION

Neurophysiological and behavioural studies have provided evidence for the modulatory effects of emotion on human memory and cognition (see Ashby, Isen, & Turken, 1999, and Fiedler, 2001, for an overview). However, we are only at the beginning to understand how emotional mood states modulate cognitive processes and neural circuits during long-term memory encoding (see Kiefer, Schuch, Schenck, & Fiedler, in press; Phelps, 2004, for a recent overview). The present research therefore investigated the neurophysiological correlates of memory encoding during good and bad emotional mood states using measurements with event-related potentials (ERPs). ERPs allow us to track the time course of brain activity during memory encoding on-line within the time range of milliseconds. ERPs are therefore well suited to study covert memory encoding processes, which do not translate into overt behaviour at this stage.

It is meanwhile well documented that emotional mood states trigger different cognitive styles, which can influence memory encoding strategies: Good mood increases creativity (Isen, Johnson, Mertz, & Robinson, 1985), productive problem solving (Isen, Daubman, & Nowicki, 1987), cognitive flexibility (Ashby et al., 1999), and knowledge-driven functions such as priming (Bless & Fiedler, 1995). Negative mood, in contrast, leads to more careful stimulus assessment (Sinclair & Marks, 1992), to decisions based on more piecemeal information search (Schwarz & Bless, 1991) and to attitudes that are more predictable from a systematic count of stimulus arguments (Mackie & Worth, 1989). Psychological theories therefore propose that good mood is associated with a creative and spontaneous cognitive style while bad mood induces a careful and controlled cognitive style (Ashby et al., 1999; Clore et al., 2001; Fiedler, 2001; Schwarz & Clore, 1988).

According to the assimilation-accommodation approach to emotion and cognition (Fiedler, 1991, Fiedler, 2001), positive mood states support assimilation, whereas negative states support accommodation. Accommodation is basically a bottom-up process by which organisms assess the environment as accurately and carefully as possible. Hence, people in bad mood exhibit a careful,inflexiblecognitivestyle. Conversely,assimilation is a top-down adaptive process by which the organism imposes its own internal structures onto the environment. This explains the creative, knowledge- driven cognitive style in people in good mood.

The notion of assimilation and accommodation functions and their relation to emotional states corresponds with the distinction of brain circuits engaged in approach and withdrawal behaviour in neuropsychological and neurobiological theories of emotion (Ashby et al., 1999; LeDoux, 1996; Rolls, 1999). The orbitofrontal cortex supposedly plays an important role in mediating reward whereas the amygdala has been suggested to be crucially involved in signalling punishment (Blood & Zatorre, 2001; Erk et al., 2003).

The assimilation-accommodation approach therefore predicts mood-dependent encoding styles (Fiedler, Nickel, Asbeck, & Pagel, 2003). Participants in good mood should be engaged in an assimilative, elaborative, semantic encoding style (e.g., forming stories out of the material to be learned). Subjects in bad mood, in contrast, should adhere to an accommodative, non-elaborative encoding style (e.g., rote memorizing; sticking to stimulus facts). Hence, successful memory should depend on different encoding strategies and correspondingly also on different neural substrates as a function of mood states. Support for this proposal comes from mood effects on memory performance within the generation effect paradigm. In the generation effect paradigm, some words in a learning list are presented completely whereas others are presented as fragments (“v - - t - ry”), and participants have to actively generate the semantic stimulus meaning (“victory”). Typically, memory for self-generated information is found to be superior to memory for passively received, experimenter-provided information (e.g., Dosher & Russo, 1976). In line with our suggestion that good mood supports an elaborative encoding style, the generation effect was larger in good than in bad mood (Fielder et al., 2003).

Within neurophysiological memory research, memory encoding processes can be studies using the so called subsequent memory effect (SME) or difference due to memory. Previous ERP studies have shown that ERPs elicited by words during encoding can be predictive of subsequent memory performance (Neville, Kutas, Chesney, & Schmidt, 1986; Paller, Kutas, & Mayes, 1987). During encoding, words that were later remembered elicited a more positive ERP than words that were not remembered between 400-700 milliseconds (ms) after stimulus onset at central and parietal electrodes. This difference between brain responses to remembered and forgotten words is referred to as SME. The scalp ERP SME can be related to findings from intracranial ERPs (Fernández et al., 1999) and fMRI studies (Erk et al., 2003; Wagner et al., 1998) in which subsequently remembered words were associated with increased activity in ventro-medial temporal areas (including fusiform and parahippocampal gyri) as well as in the inferior frontal cortex (see Paller & Wagner, 2002, for an overview). These brain regions are also known to be involved in semantic processing (e.g., Vandenberghe, Price, Wise, Josephs, & Frackowiak, 1996). The precise nature of the processes which give rise to the SME is poorly understood (Paller & Wagner, 2002). In support of the view that semantic processes contribute to successful memory encoding (Craik & Lockhart, 1972), the SME is larger in semantic than in non-semantic encoding tasks (Paller et al., 1987). However, the processes underlying the SME are probably not confined to semantic processes and other presently unknown processes also contribute (Fernández et al., 1998).

A recent fMRI study shows that the SME depends on the emotional context during encoding. Erk et al. (2003) investigated the influence of the emotional context (positive, negative, and neutral emotional pictures) on the encoding of words using fMRI. Within a positive emotional encoding context activity to subsequently recalled words was greater in parahippocampal and fusiform gyri than to forgotten words (SME). As mentioned above, these areas play also an important role in semantic processing. Within a negative context, in contrast, SME-related activity was greater in the right amygdalar region, an area involved in signalling punishment and in fear processing (LeDoux, 1996). The common involvement of brain areas in semantic processing and in successful episodic memory encoding (see also Lepage, Habib, Cormier, Houle, & McIntosch, 2000), particularly in a positive emotional context, is in line with the suggestion of the assimilation-accommodation approach that good mood supports activation of semantic knowledge structures during episodic memory encoding. The study by Erk et al. (2003) assessed the influence of a strong and rapidly changing emotional encoding context (i.e., high arousing emotional pictures), but the influence of subtle and long-lasting mood states on the neurophysiological correlates of memory encoding have not been determined yet. 

In the present study, we therefore tested the assumption of mood-dependent episodic memory encoding processes by investigating the influence of participants’ mood state on the SME. Before memory encoding, separate groups of our participants viewed funny or sad movies to induce good and bad mood states, respectively. ERPs were recorded while participants in these different groups were presented with words that had to be memorized for subsequent recall. Words could be either complete or had to be actively generated from fragments. We manipulated the nature of the encoding task, because the influences of the mood state were expected to be largest for the generated words, an encoding task which requires an assimilative encoding style. ERPs to encoded stimuli were sorted according to whether the words were later recalled or not in order to determine the SME. As good mood supports semantic encoding styles and as the magnitude of the SME has been shown to be increased during semantic compared with non-semantic encoding, we expected the SME to be larger in good than in bad mood, particularly in the generative encoding task.

## Method

### Participants

Thirty-eight right-handed volunteers (8 male, 30 female; mean age 26 years) with normal or corrected-to-normal vision participated in the study. Handedness was assessed with the Oldfield Handedness Inventory (Oldfield, 1971). Participants were native German speakers without any history of neurological or psychiatric illnesses. Gender was identically distributed (15 female and 4 male) in the two participant groups who received an induction of a good or bad mood state, respectively (for the mood induction procedure see below). All participants signed a written consent after the nature and the consequences of the experiment had been explained. When providing information about our study, participants received a cover story that contained all elements of the experimental procedure, but concealed the true purpose (for a detailed description of the instructions, see the Material and Procedure section). We used a cover story, in order to minimize the possibility that participants would discover our experimental hypotheses. The study has been approved by the local Ethical Committee.

### Material and procedure

As stimuli served 160 adjectives referring to positive and negative personality traits. All 160 words were fragmentized by removing one to three letters from each word, depending on word length. It was controlled that word fragments were unequivocal. The first letter of a word was never removed. Adjectives were divided into eight lists of 20 words each. Each list contained an equal number of complete and fragmentized word stimuli. Mean word frequency and word length were matched across lists. Stimulus order within each list and presentation order of lists were randomized. For each word list were created versions A and B. When a word was presented in its complete form in version A, it was presented as a fragment in version B, and vice versa. The A and B versions of the lists, as well as list order, were counterbalanced across participants. Thus, across participants each word appeared equally often in the complete and the fragmentized form, respectively.

Participants were seated in front of a computer screen in a dimly lit, electrically shielded, sound attenuated booth. They were instructed that the study was aimed at revealing the influence of mental work load due to a memory task on mood. They were told that during the study several films would be shown, and that they were supposed, for the success of the study, to let the films take effect on them emotionally. Participants were also informed that they would be presented with eight word lists which had to be memorized and recalled. Stimuli were displayed in white font against a black background in the centre of a computer screen synchronously with the screen refresh rate. Participants were first presented with a fixation cross for 750 ms, thereafter with a word from the list for 1200 ms, which could be complete or fragmented. Subsequently, a blank screen was shown for 1800 ms. Then, a question mark appeared for 2000 ms which prompted the participants to name the word aloud. They had to withhold the response until to the appearance of the question mark in order to avoid movement-related artefacts in the EEG. The produced name and the correctness of the response were recorded by the experimenter. After the question mark disappeared, a hash mark was presented for 2700 ms to signal the participants the intertrial interval (ITI). The next trial started again with the presentation of the fixation cross. After the presentation of each list, a free recall test was performed. Participants had to orally recall as many words as possible of the immediately learned list within three minutes. The experimenter noted all produced words and classified them later as being correctly recalled or not.

At the beginning of the experiment, participants were familiarized with the procedure. Thereafter, a first mood rating using a visual analogue scale with the anchors very *depressed* and very elated was administered. Afterwards, mood states were induced by showing the first film. Half of the participants were induced with a good mood (funny films), the other half with a bad mood (sad films). After film presentation, they had to rate their current mood again. Thereafter, participants had to learn and to recall the first and afterwards the second word list. This cycle of mood induction, manipulation check, learning and recall of two lists was repeated four times. At the end of the experiment, participants were asked for the strategies they had employed for memorizing the words. Finally, participants were debriefed. An entire experimental session including electrode placement took about three hours.

### EEG-recording and signal extraction

Scalp voltages were recorded using an equidistant montage of 64 sintered Ag/AgCl-electrodes mounted in a cap (Easy Cap, EasyCap, Herrsching, Germany). An electrode between Fpz and Fz was connected to the ground, and an electrode between Cz and FCz was used as recording reference. Eye movements were monitored with supra- and infra-orbital electrodes and with electrodes on the external canthi. Electrode impedance was kept below 5 kΩ. Electrical signals were amplified (70 Hz-DC, 50 Hz notch filter), continuously recorded (digitization rate = 250 Hz), digitally band-pass filtered (high cut-off: 16 Hz, 24 dB/octave attenuation; low cut-off: 0.1 Hz, 12 dB/octave attenuation) and segmented (150 ms before to 800 ms after the onset of the word to be encoded). Artefacts from vertical eye movements and eye blinks were removed according to the regression technique, suggested by Gratton, Coles, and Donchin (1983). EEG segments were baseline-corrected to the 150 ms pre-stimulus interval. Segments exceeding a potential threshold of +/- 75 µV were rejected as artefacts. Segments with correct naming responses during encoding were averaged separately for subsequently recalled and not recalled words. ERP data analysis was performed using the BrainVision Analyzer (BrainProducts, Gilching, Germay). In order to obtain a reference-independent estimation of scalp voltage, the average-reference transformation was applied to the ERP data (Bertrand, Perrin, & Pernier, 1985; Kiefer, Marzinzik, Weisbrod, Scherg, & Spitzer, 1998). Due to the limited amount of available trials, ERPs to positive and negative trait words, which were comparably distributed in the recalled and not recalled conditions, were pooled to determine the SME. ERP effects of the positive and negative trait words independent of subsequent memory are reported in a companion article (Kiefer et al., in press).

## Results

### Manipulation check of mood induction:

Mood ratings before the first film (*t*_0_) and after the four mood inducing films (*t*_1_...*t*_4_) were submitted to a repeated-measures ANOVA with time point of rating (*t*_0_...*t*_4_) as within-subject factor and induced mood state as between-subject factor. As expected, this analysis yielded an interaction between both factors, *F*(4, 36) = 15.3, *MSE* = 165, ε = .592, *p* < .0001. Post-hoc tests showed that mood ratings did not differ between groups at *t*_0_, before the mood induction, but at all of the subsequent time points. Participants receiving the sad movies (bad mood condition) rated their mood as being more depressed compared with participants receiving the funny movies (good mood condition). Hence, the mood induction procedure was successful and produced changes of mood ratings in the expected direction.

### Encoding task

Performance in the encoding tasks was close to ceiling in all participants. Participants named on average 39.53 words (*SD* 0.61) correctly (out of 40) in the “read” condition and 39.11 words (*SD* 0.69) in the “generate” condition.

### Free recall

A repeated-measures ANOVA with encoding task (generate vs. read) and mood state (good vs. bad) as between-subject factor was performed on free recall rate (percentage of correctly recalled words). A significant effect of encoding task, *F*(1, 36) = 13.3, *MSE* = 0.51, *p* < .001, showed that generated words (39.5%) were better recalled than read words (35.3%). As expected, memory performance was highest (40.8%) when participants in good mood recalled generated words (bad mood: 38.3%). However, the interaction between mood and encoding task did not reach significance.

### Encoding strategy

Reported encoding strategies were classified as either elaborative (e.g., forming a story out of the presented words, associating the words with specific persons), non-elaborative (e.g., list rehearsal, rote memorizing), or a combination of both. A chi-square test revealed a significant association of strategy choice and mood state, χ^2^(2) = 9.8, *p*< .01. Participants in good mood reported most frequently elaborative strategies (15 out of 19 participants), participants in bad mood most frequently non-elaborative strategies (8 out of 19 participants).

### Electrophysiological results

**Figure 1.**
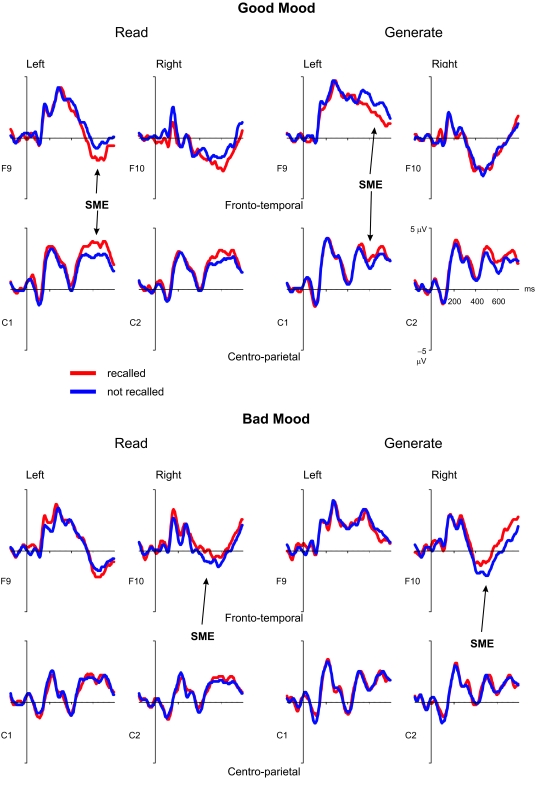
Grand averaged ERPs of participants in good mood (A) and bad mood (B) from selected electrode positions according to the standard 10/20 system (fronto-temporal: F9/F10; central: C1/C2) as a function of subsequent recall and encoding task. Negativity is plotted down.

Figure 1 shows that recalled words elicited a more positive potential (between 500-700 ms over the parietal and central scalp) than words which were not recalled. During the same time interval, a polarity-reversal was found over fronto-temporal areas where recalled words elicited a relatively greater negative potential than not recalled words.

Mean voltages were analyzed statistically in the time window from 500-650 ms after stimulus onset, in which the SME is largest (Neville et al., 1986; Paller et al., 1987). Three scalp regions of interest, each of them being represented by three pairs of contra-lateral electrodes, were selected for analysis: occipito-parietal (O1/O2, PO3/PO4, P1/2), centro-parietal (CP3/CP4, C1/C2, C3/C4) and fronto-temporal (T1/T2, FT7/FT8, F9/F10). Voltages were collapsed across electrode sites. The centro-parietal electrode sites were chosen because the SME is known to be largest in this scalp region (Neville et al., 1986; Paller et al., 1987). As ERP effects related to semantic processing have been reported over fronto-temporal scalp electrodes (Kiefer, Weisbrod, Kern, Maier, & Spitzer, 1998; Snyder, Abdullaev, Posner, & Raichle, 1995), this region was also selected. Repeated-measures ANOVAs with the within-factors recall (recalled vs. not recalled), encoding task (generate vs. read) and hemisphere and the between-factor mood state (good vs. bad mood) were performed separately for each scalp region (*p*= .05). Significant interactions were further evaluated with Fisher LSD post-hoc tests.

ERPs to subsequently recalled and forgotten words significantly differed at centro-parietal electrodes: A main effect of recall, *F*(1, 36) = 7.3, *MSE* = 3.70, *p* < .05, indicated that recalled words elicited a more positive potential than not recalled words (SME). The SME tended to be larger in good than in bad mood, interaction mood x recall: *F*(1, 36) = 3.2, *MSE* = 3.70, *p* = .081. In fact, as it can be seen from figure 1, the central SME was almost absent for participants in bad mood.

At fronto-temporal electrodes, a polarity-reversed SME was observed: Potentials to recalled words were less positive than to not recalled words. A significant recall x mood interaction indicated that the fronto-temporal SME differed between mood states, *F*(1, 36) = 4.1, *MSE* = 8.32, *p* < .05. Separate ANOVAs were performed for the mood states in order to evaluate this interaction. In good mood, a main effect of recall was obtained, *F*(1, 18) = 5.2, *MSE* = 10.17, *p* < .05: Potentials to recalled words were less positive than to not recalled words. In bad mood, the recall X hemisphere interaction was significant, *F*(1, 18) = 4.8, *MSE* = 2.06, *p* < .05. This interaction was due to the fact that over the right hemisphere potentials to recalled words were more positive than to not recalled words. Hence, in contrast to participants in good mood, participants in bad mood did not exhibit a fronto-temporal SME, which was polarity-reversed compared with that at parietal and central scalp regions. Instead, they showed a right lateralized SME of the same polarity as the central SME.

**Figure 2.**
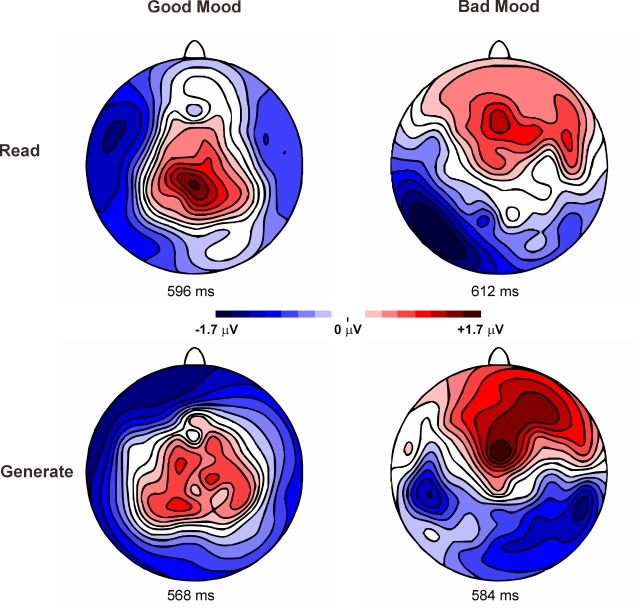
Topography of the subsequent memory (SME) ERP effect as a function of mood state and encoding task. Shown is the potential distribution of the difference waves (recalled minus not-recalled words) interpolated across the head using spherical splines. The maps are taken at the maximum SME in the time window of 500-650 within each condition. The scalp distribution of the SME is indicated by positive voltages (red colour).

At occipito-parietal electrodes, no effect involving the factor recall reached significance. The scalp distribution of the SME as a function of mood states is shown in Figure 2.

## Discussion

Using ERP measurements, we asked whether the neuro-cognitive mechanisms underlying successful memory encoding depend on the emotional mood state. To this end, we assessed for the first time whether the SME, an electrophysiological index for successful stimulus encoding, is modulated by participants’ current mood state. Analysis showed that potentials to subsequently recalled words were more positive than to not recalled words at centro-parietal electrodes in the time interval of 500-650 ms after stimulus onset. This ERP difference due to subsequent memory is consistent with earlier demonstrations of the SME (e.g., Fernández et al., 1998; Neville et al., 1986; Paller et al., 1987).

Most importantly, the SME varied as a function of mood state: The centro-parietal SME tended to be larger in good than in bad mood. The strongest differences in SME between subject groups were obtained at fronto-temporal electrodes: Participants in a good mood showed a bilateral SME, which was polarity-reversed compared with that at centro-parietal electrodes. Participants in a bad mood, in contrast, exhibited a SME over the right fronto-temporal scalp exhibiting the same polarity as the centro-parietal SME. These differences in the topography of the SME suggest that successful subsequent recall relies on partially different neural circuits during encoding for good and bad mood states.

We propose that mood states support different encoding processes that contribute to later successful recall. According to the assimilation-accommodation approach (Fiedler, 2001; Fiedler et al., 2003), in good mood stimuli are encoded by transforming incoming information on the basis of stored knowledge structures whereas in bad mood information of stimuli to be encoded is more likely conserved, with no or only minor transformation. Consistent with this assumption, participants in good mood most frequently reported to have applied elaborative semantic encoding strategies, such as forming stories out of the words, whereas participants in bad mood most frequently mentioned to have used non-elaborative strategies such as rehearsal of word lists. Although the present results are entirely compatible with the assimilation-accommodation approach, they can also be accounted for by other theories, which propose an association between emotional mood states and cognitive styles (e.g., Ashby et al., 1999; Clore et al., 2001).

As good mood supports assimilative processing, the generation effect (superior memory performance for word generation, an assimilative encoding task) should be enhanced in good mood. In contrast to our predictions and to previous findings (Fiedler et al., 2003), this memory advantage for self-generated material was not significantly larger in good than in bad mood, although recall rates showed numerically the expected pattern. Likewise, the SME in good mood was not larger for word generation. Instead, the magnitude of the SME did not significantly differ between the encoding conditions.

The missing interaction between mood and encoding task could be explained by three factors: Firstly, participants in good mood applied elaborative encoding strategies (i.e., formed stories out of the words), irrespective of whether the words were read or generated (see also below). This could have eliminated the additional influence of the manipulation of the encoding task within the experiment. Secondly, the long and straining recording session with eight repetitions of list learning and recall could have induced proactive interference from previous lists thereby deteriorating memory performance and increasing random noise in recall performance. In fact, recall performance was relatively low, and the generation effect was atypically small in our study. Thirdly, encoding of the fragmented words could have produced an increased latency jitter of the encoding processes reflected in the SME across the different trials because encoding difficulty of the word fragments could vary more strongly than difficulty of the intact words. This latency jitter could have smeared the signal in the averaged ERP and decreased the amplitude of the SME.

A fronto-temporal SME polarity-reversed compared with that at centro-parietal electrodes, as observed in our participants in good mood, has not been reported previously. This is probably due to the fact that the average-reference transformation was not applied to the previous data. It is possible that the fronto-temporal and the central SME are generated by the same brain tissue (probably ventro-medial temporal structures) and electrical currents are simply volume-conducted to different partitions of the scalp. The involvement of the ventro-medial temporal lobe (fusiform and parahippocampal gyri) in generating the SME has been demonstrated in previous studies with intracranial ERP recordings (Fernández et al., 1999) as well as in neuroimaging studies (Erk et al., 2003; Wagner et al., 1998). Alternatively, the fronto-temporal SME could specifically reflect elaborative encoding, possibly in inferior frontal cortex (Wagner et al., 1998). Future research is needed to clarify the significance and the neural generators of the fronto-temporal SME. Irrespective of the definitive answer to this question, our data demonstrate that successful memory encoding depends on differential neural pathways as a function of the emotional mood state.

The present results are compatible with those from a recent fMRI study on memory encoding during rapid changes of different emotional contexts (Erk et al., 2003). In this fMRI study, SME related activity in the right amygdalar region was observed in an emotionally negative encoding context. SME related activity for words encoded within a positive emotional context, in contrast, was greater in parahippocampal and fusiform gyri. Of course, the localizational value of ERPs has to be viewed with caution so that the effects on scalp ERPs cannot be directly related to activity differences in particular brain structures (Nunez, 1981). Nevertheless, the present study confirms and extends these earlier findings by showing that even subtle long-lasting mood states modulate the way how stimuli are successfully encoded into memory. In the Erk et al. study, the emotional context was provided by high arousing positive and negative pictures immediately presented before the word to be encoded. Moreover, the emotional valence of the context changed rapidly. Hence, in the Erk et al. research emotional stimulation presumably yielded strong, but short-lived affective states. In our study, in contrast, we induced subtle emotional states by presenting movies several minutes before the word encoding task. This procedure resulted in diffuse mood states that last for a relatively long time interval. Despite these differences in the precise characteristics of the emotional state, a modulation of the SME was obtained in both studies suggesting that a relative general emotional mechanism is involved.

In the subject sample assessed in our study, the broad majority of participants were female. However, as gender was identically distributed in the groups with good and bad mood states, respectively, it can be ruled out that our observation of mood-dependent memory encoding was compromised by gender effects. Of course, this does not preclude the possibility of gender differences in emotional memory although previous results are highly contradictory in this respect (see also Rolls, 1999). For instance, sometimes women were more susceptible to emotional stimulation than men (Bremner et al., 2001). Nevertheless, other authors did not find any gender differences in emotional sensitivity (Piefke, Weiss, Markowitsch, & Fink, 2005). Likewise, there are reports of more activity in emotion-related brain regions in women than in men (Bremner et al., 2001; Canli, Desmond, Zhao, & Gabrieli, 2002), as well as of gender-related differences in hemispheric-specific activation of the amygdala (Cahill et al., 2001; Canli et al., 2002) and prefrontal cortex (Piefke et al., 2005) during emotional memory tasks. However, a further study found less emotion-related amygdalar activity in women than in men (Schneider, Habel, Kessler, Salloum, & Posse, 2000). Hence, available evidence on gender differences in emotional processing is rather mixed and does not convey a consistent picture. As this issue was not central to the aim of our study, and as our sample included only four male participants in each group, gender effects were not further evaluated.

We assume that our present findings can be explained best on the grounds of differential encoding strategies as the assimilation-accommodation account suggests: Good mood promotes the active transformation of new information by applying existing semantic knowledge to incoming information in order to achieve a coherent memory structure (assimilation). Bad mood, in contrast, supports non-elaborative encoding like rote rehearsal without the active application of semantic knowledge to the incoming information. Accordingly, in bad mood the new information is changed very little during encoding so that episodic memory structure has to be altered to fit the new information (accommodation). As a result, participants in a good mood are more likely to employ deep semantic encoding strategies (Craik & Lockhart, 1972) in comparison to participants in a bad mood.

It is open through which precise neuro-cognitive mechanism emotional states trigger cognitive styles and encoding strategies. Possibly, emotionally positive situations – subtle mood states as in the present study or salient emotional stimuli – signal reward by activating brain circuits such as orbitofrontal cortex and the dopaminergic neurons passing through the nucleus accumbens and projecting to prefrontal cortex and the anterior cingulate. Ashby et al. (1999) propose that the creative and elaborative cognitive style in good mood is the result of dopaminergic neuromodulatory action on neurons in the anterior cingulate, which improves cognitive flexibility by facilitating executive attention. Emotionally negative situations signal punishment and might influence cognition through neural pathways involving the amygdala and prefrontal cortex, which are reciprocally connected (Ghashghaei & Barbas, 2002). Possibly, activity in the amygdala down-regulates processing in prefrontal structures (Drevets & Raichle, 1998; Siegle, Thompson, Carter, Steinhauer, & Thase, 2007). This would explain why negative emotional situations deteriorate cognitive flexibility and induce an accommodative encoding style. 

We are aware that our study represents only a first step in investigating mood influences on the SME. Therefore, the proposed relation between mood, cognitive styles and memory should be further tested in future studies. For instance, the assimilation-accommodation factor could be operationalized by contrasting encoding tasks which are more “pure” with respect to this distinction than the presently used generation effect paradigm. In particular, the read condition of the generation paradigm offers participants many degrees of freedom for differential encoding strategies, which cannot be properly controlled. One might therefore assess memory performance and the SME for a perceptual (e.g., letter search) and a semantic encoding task (e.g., semantic categorization, fragment completion) as a function of participants’ mood. The perceptual encoding task affords an accommodative encoding strategy in a more constrained way than the reading task. The assimilation-accommodation account predicts superior memory performance and an enhancement of the SME in good mood in comparison to bad mood, but only for the assimilative semantic encoding task. For the accommodative perceptual encoding task, in contrast, memory performance and SME should be comparable for good and bad mood. Instead of varying the differential contribution of assimilation and accommodation to encoding, one might vary contribution of these factors to memory retrieval. While free recall is a retrieval task with a strong assimilative component because the retrieval cues have to be self-generated, recognition memory emphasises accommodation and assimilation similarly because the retrieval cue is experimenter-provided. As good mood supports assimilative retrieval processes, memory performance and amplitude of the SME during encoding should be greater for good than for bad mood, particularly in the free recall task.

In conclusion, our study shows for the first time that the neuro-cognitive mechanisms during encoding subserving later successful recall depend on the emotional mood state. Participants in good mood reported to have more frequently applied elaborative encoding strategies in comparison to participants in bad mood. At a neurophysiological level, we found that the ERP SME was larger in participants in good than in bad mood. Furthermore, the SME exhibited a different topography for the different mood states over fronto-temporal areas suggesting that different brain structures are involved. The present results demonstrate that subsequent successful recall is established by differential neural pathways and cognitive processes depending on subtle long-lasting mood states. Our findings are in line with the view that emotional mood states influence memory encoding by triggering different encoding styles.

## Acknowledgements

Supported by grants from the University of Ulm Medical School (P.506, P.638) and from the German Research Community (DFG Ki 804/1-1, DFG Ki 804/1-3) to M.K. The authors thank Tatjana Zimmermann for her help during data acquisition and two reviewers for helpful comments on an earlier version of this manuscript.
